# Prehospital Management of Gunshot Patients at Major Trauma Care Centers: Exploring the Gaps in Patient Care

**DOI:** 10.5812/traumamon.10438

**Published:** 2013-08-14

**Authors:** Amir Norouzpour, Ali Reza Khoshdel, Mohammad-Hadi Modaghegh, Gholam-Hossein Kazemzadeh

**Affiliations:** 1 Department of Epidemiology, Faculty of Medicine, AJA University of Medical Sciences, Tehran, IR Iran; 2 Vascular and Endovascular Surgery Research Center, Imam Reza Hospital, Mashhad University of Medical Sciences, Mashhad, IR Iran

**Keywords:** Wounds, Gunshot, Triage, Emergency Medical Services, Wounds and Injuries, Iran

## Abstract

**Background:**

Prehospital management of gunshot-wounded (GW) patients influences injury-induced morbidity and mortality.

**Objectives:**

To evaluate prehospital management to GW patients emphasizing the protocol of patient transfer to appropriate centers.

**Patients and Methods:**

This prospective study, included all GW patients referred to four major, level-I hospitals in Mashhad, Iran. We evaluated demographic data, triage, transport vehicles of patients, hospitalization time and the outcome.

**Results:**

There were 66 GW patients. The most affected body parts were extremities (60.6%, n = 40); 59% of cases (n = 39) were transferred to the hospitals with vehicles other than an ambulance. Furthermore, 77.3% of patients came to the hospitals directly from the site of event, and 22.7% of patients were referred from other medical centers. EMS action intervals from dispatchers to scene departure was not significantly different from established standards; however, arrival to hospital took longer than optimal standards. Additionally, time spent at emergency wards to stabilize vital signs was significantly less in patients who were transported by EMS ambulances (P = 0.01), but not with private ambulances (P = 0.47). However, ambulance pre-hospital care was not associated with a shorter hospital stay. Injury Severity was the only determinant of hospital stay duration (β = 0.36, P = 0.01) in multivariate analysis.

**Conclusions:**

GW was more frequent in extremities and the most patients were directly transferred from the accident site. EMS (but not private) ambulance transport improved patients' emergency care and standard time intervals were achieved by EMS; however more than a half of the cases were transferred by vehicles other than an ambulance. Nevertheless, ambulance transportation (either by EMS or by private ambulance) was not associated with a shorter hospital stay. This showed that upgrade of ambulance equipment and training of private ambulance personnel may be needed.

## 1. Background

Trauma is the fourth leading cause of death in Iran, and nearly 15.2% of all deaths seem to be due to trauma (1, 2). Gunshot trauma (GW) is a virtually lethal trauma, and it is considered as one of the leading causes of mortality in developing countries (3). Gunshot-induced injuries of different body parts (e.g., head, spine, chest and vessels) and medical approaches to them have been reported in the literature (4-7).Beside the medical importance, gunshot trauma imposes a high economic load on societies, and it considered as one of the four expensive causes of hospitalization in the United States (8, 9).The dispatch rate of emergency medical service (EMS) varies widely in different regions and depends on several socioeconomic factors as well as accessibility of emergency care centers (10). Additionally pre-hospital emergency care includes immediate assessment and response, timely transport and appropriate plan for admission. Many studies have shown that "organized trauma systems" reduce mortality rates (11-13). The results of such studies have sparked concerted efforts to establish trauma systems throughout the United States and some other developed countries over the past three decades. Strong emphasis has been given to the improvement of the emergency medical services (EMS) systems which play a critical role in every trauma system (14, 15). Several studies suggest that rapid transport rather than prolonged in-scene treatment should be given the highest priority (16). In particular, priorities for trauma system improvement in developing countries should focus on more rapid prehospital transport and improved en route care (17). Such improvements would likely decrease overall mortality, and would also be less expensive than enhancing expensive intensive care capabilities and other hospital-based technologies.

## 2. Objectives

A few studies demonstrated higher mortality of EMS-transported patients compared to non-EMS vehicles even after adjustment for injury severity (15). However, there are little studies assessing the impact of prehospital emergency care, particularly in GW patients; in this study, we evaluate prehospital approaches of a trauma system to GW patients focusing on the triage and transport vehicles of patients to the hospitals.

## 3. Patients and Methods

This prospective study from March 2010 to March 2011, was conducted at four major level-I hospital centers in Mashhad, Iran. All GW patients came to the hospitals for treatment. A specific data collection form was used. Our data included demographics of the injured person, the site of event, the source of accident (homicide, suicide, or accidental), injured body parts, triage and transport vehicles used, the dispatch time of EMS ambulance, vital signs and Glasgow Coma Scale (GCS) of injured at the time of arrival to the hospitals, hospitalization time of patients and the eventual outcome (discharge from the hospitals or death). Severity of injury was measured by the Injury Severity Score (ISS). To compute the ISS of each patient, the body regions of an injured were defined as: head and neck, face, thorax, abdomen, extremities and skin. The injury severity of each body region was quantified on the basis of clinical judgment. Thereafter, the squares of three greatest numbers were added to yield the ISS. All statistical analyses which included Mann-Whitney Test, Spearman Correlation, Kruskal-Wallis Test, and Chi-Square Test were done using SPSS software (v. 11.5). Confidence interval of 95% and a level of significance of P < 0.05 was considered for all statistical tests.

## 4. Results

**Figure 1.**
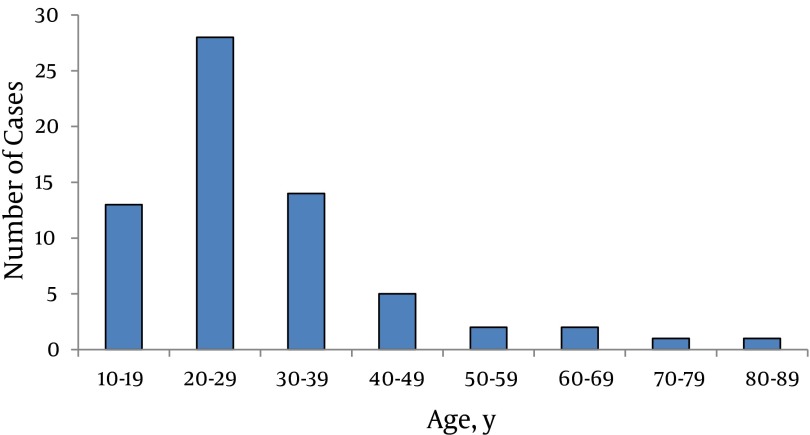
Age Distribution of the Patients

Total of 66 GW patients referred to the hospitals during a one year period. Sixty one patients (92%) were male. Patients’ age ranged from 10 to 82 years (Figure 1), 28 patients (42%) were 20-29 years old, 14 patients (21%) in the age group of 30-39 years, and 13 patients (20%) in the age group of 10-19 years.

The most common cause of GW was homicide (74%, n = 49), 23% of cases (n = 15) were injured accidentally, and 3% (n = 2) were injured when attempting suicide.

**Figure 2.**
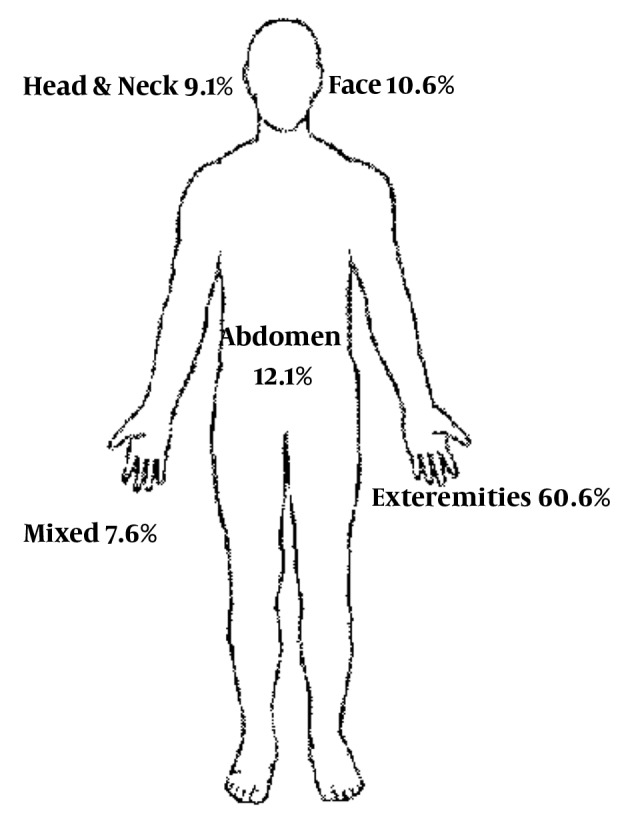
Distribution of the Patients on the Based on the Injured Body Regions

The most common affected body parts were extremities (60.6%, n = 40), followed by abdomen (12.1%, n = 8) and face (10.6%, n = 7). Distribution of the injured body regions are shown in Figure 2.

**Figure 3.**
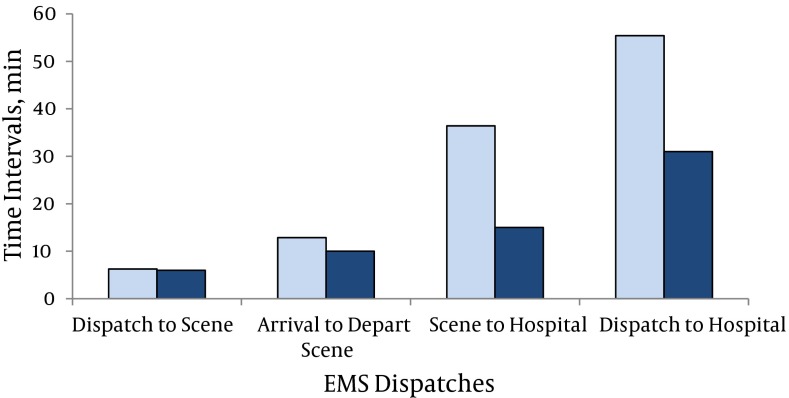
Different Time Intervals of EMS Dispatches Dark blue bars: Standard; Light blue bars: This study

Data recorded on the triage of patients showed that 77% of cases (n = 51) came to hospitals directly from the site of the event in the city (74%, n = 49) or suburbs (3%, n = 2); 23% of cases (n = 15) were referred to the hospitals from other centers which included: 3% (n = 2) from centers in which the patients were admitted, 12% (n = 8) from centers to which the patients were transferred only to receive primary care and 8% (n = 5) from forensic centers. About 20% of cases (n = 13) were transferred to the hospitals with EMS ambulances. They included 12 cases transferred directly from the site of event in the city, and one case transferred from a forensic center. However, 21% of cases (n = 14) were transferred to hospitals with non-EMS ambulances. They included 2 cases referred from centers in which they were admitted, 8 cases referred from centers to which they were transferred only for receiving primary care, and 4 cases referred from forensic centers. Therefore, 59% of cases (n = 39) were transferred to the hospitals without an ambulance. They included 37 cases that came directly from the site where the injury occurred in the city, and 2 cases came directly from the site of event occurred outside of the city (Figure 3).

•The mean of the dispatch-beginning-to-scene-arrival interval was 6.25 minutes (ranged from 3 to 13 minutes), not significantly different from standard of 6 (P = 0.811).

•The mean of the time interval spent by EMS personnel at the site of event was 12.85 minutes (ranged from 4 to 20 minutes), not different from standard of 10 (P = 0.051).

•The mean of the scene-departure-to-hospital-arrival interval was 36.42 minutes (ranged from 5 to 93 minutes), significantly longer than standard of 15 (P = 0.008).

•The mean dispatch-beginning-to-hospital-arrival interval was 55.42 minutes (ranged from 25 to 114 minutes) which was longer than standard of 31 (P = 0.015).

Upon arrival to the hospitals, the mean systolic blood pressure of patients was 113 ± 15 (ranged 70-190 mmHg), whereas that of diastolic blood pressure was 72 ± 8 (ranged 40-90 mmHg). The mean of pulse rate of patients at this time was 84±13 beats/min (ranged 38-120 beats/min). The mean respiratory rate of patients was 16 ± 3 per minute (ranged 7 - 30). GCS of most cases (95%) was ≥ 14. The range of GCS in our series was 3-15. GCS of patients was significantly correlated with their blood pressure at the time of arrival to the hospitals (P = 0.001). Moreover, Spearman Correlation test demonstrated an inverse relationship between systolic blood pressure and the injury severity score (ISS) (rho = -0.29, P = 0.019). There was no difference in vital signs in the subgroups of transport system (P > 0.05).

**Figure 4.**
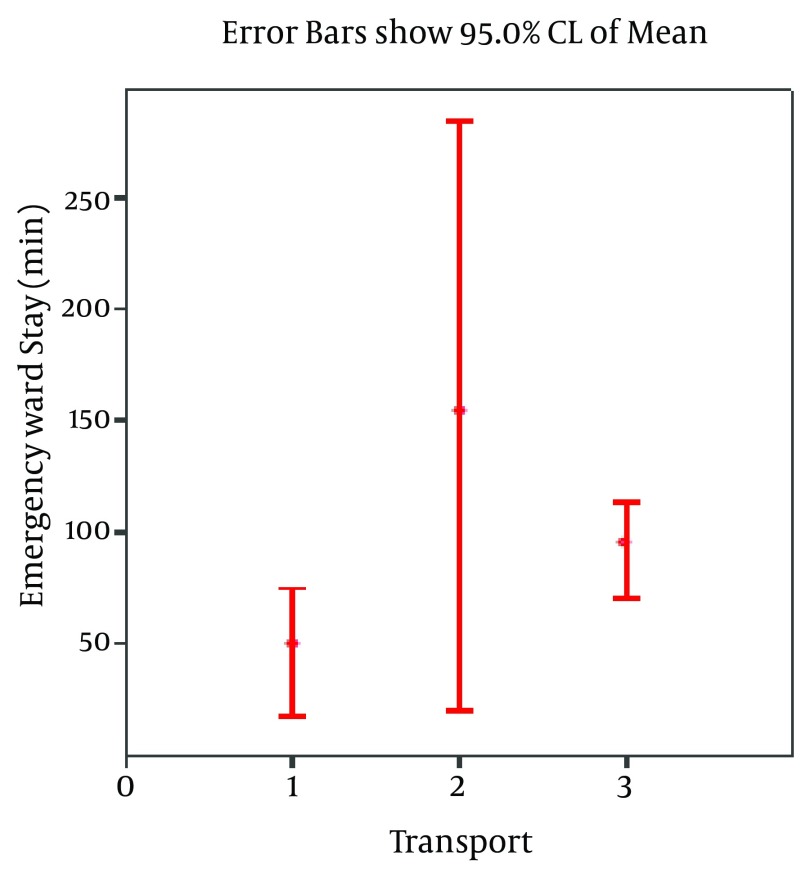
Duration of Emergency Ward Stay of Patients Based on Type of Transport to the Hospitals 1: EMS ambulance; 2: private ambulance; 3: non-ambulance vehicle

After arrival to the hospitals, patients received emergency care until their vital signs remained stable. Non-parametric tests demonstrated that EMS-ambulance transported patients had a significantly shorter stay in emergency ward than the group transported by non-ambulance vehicles (median 22 minutes *versus *74 minutes, respectively; P = 0.01). There was not such a difference for private ambulance-transported group (P = 0.47) (Figure 4).

Our results showed that, 5 cases were injured mildly (ISS < 7), 7 cases injured moderately (ISS was from 7 to 12), and 1 case injured severely (ISS > 12); of the 66 patients, 6 cases left the hospital before they were formally discharged. Of the remaining 60 patients, 57 cases were discharged from the hospital without any permanent complication, and 3 cases expired. The mean hospitalization time of patients who were eventually discharged from the hospital was 6 days (ranged from 0 to 39 days). Analyses showed that, although patients who were referred from other centers had longer hospitalization time than those that came to the hospitals directly from the site of event, it was not significantly different (P = 0.731). In respect to the hospitalization time on the basis of patients’ triage, patients who were referred from forensics had the longest hospitalization time ( mean = 13 days). However, the hospitalization time was not significantly different among the triage-based groups (P = 0.188). ISS was also associated with hospitalization duration (P = 0.03). But, the hospitalization time was not significantly affected by transport vehicles of patients to the hospitals in general (P = 0.467). In order to eliminate the confounders, we decided to evaluate only the patients who came to the hospitals directly from the site of event, only in the city. As a result, with a comparable ISS (P = 0.72), patients who were transferred with EMS ambulances stayed in the hospital for a shorter period of time than those transferred with non-ambulance vehicles (P = 0.017); however there was no significant difference between transport by non-EMS with the other two groups. Multivariate analysis with age, transport group, vital signs and injury severity demonstrated that ISS was the only determinant of hospital stay duration (β = 0.36, P = 0.01).

## 5. Discussion

### 5.1. Triage of Patients

 In an ideal trauma system, different centers providing medical services of different levels are matched to each injured patient on the basis of the medical services needed (18). The need of each injured person to the level of medical services are evaluated at the scene of the incident and the patient is triaged based on vital signs and the severity of injury. Therefore, an injured case who has critical condition is promptly transferred to an appropriate center to receive the required medical services as soon as possible, preventing deterioration of the patient’s condition. On the other hand, it prevents transferring of an injured patient in relatively good condition to a high-level center, decreasing patient load of such centers. In our study, patients who were referred from other centers stayed in the hospitals for a longer period of time than those that came directly from the site of the incident. However, the hospitalization time of triage-based groups showed that patients referred from forensic centers stayed longer than other groups. Although they had higher ISS than others, another factor which may affect the hospitalization time is the time of delays for transferring the patients to the hospitals. In our series, these patients were transferred from the site of event to forensic centers, and then to the hospitals.

### 5.2. Transport Vehicles of Patients

There is a considerable diversity in use rate of EMS in various regions even in a country which is associated with socioeconomic dilemmas and less accessibility to emergency care centers (10). In an ideal trauma system, providing the medical services to patients begins from the time at which an accident occurs (18). Transport vehicles of patients should also be equipped to provide medical services to patients during the transportation to a hospital. This may prevent deteriorating of patients’ conditions. Our results showed that the emergency ward stay (in the whole group) and hospitalization time (within the city) of GW patients who came to the hospitals directly from the site of event with EMS ambulances was significantly different from that of patients came with non-ambulance vehicles. However, given a comparable injury severity in the subgroups, it seems that immediate care by private-ambulances was not so efficient and a continued education for ambulance staff is necessary. Another issue is the importance of providing more accessible EMS both inside and outside the city. Although our small sample size affects the results, it is obvious that more speculation on factors which may affect EMS performance can improve the trauma system more efficiently. Such factors may include ambulance equipment, as well as knowledge and experience of ambulance personnel, particularly in private services. These must include enhancement of knowledge and equipments for a more efficient practice that could reduce hospitalization rate and duration. Other studies are needed to recognize such factors, through which potential points for EMS improvement may be shown.

### 5.3. Time Intervals

Transportation plays a major role during the golden time for patients care. Many studies suggest that rapid transport rather than prolonged in-scene treatment should be given the highest priority ( 16 ). In our study, the mean dispatch-beginning-to-scene-arrival interval was 6.25 minutes (Figure 3, Table 1).

**Table 1.** Different Time Intervals of EMS Dispatches

Time Intervals	Mean, min	SD, min	Minimum, min	Maximum, min
**Dispatch-Beginning-to-Scene-Arrival**	6.25	3.60	3	13
**Scene-Arrival-to-Scene-Departure**	12.75	4.92	4	20
**Scene-Departure-to-Hospital-Arrival**	36.42	26.20	5	93
**Dispatch-Beginning-to-Hospital-Arrival**	55.42	29.47	25	114

Compared with the mean of the accident-happening-to-scene-arrival interval of a given developed EMS system such as Seattle (i.e., 6 minutes) (19), ours seems acceptable. It seems that favorable geographical distribution of the EMS units throughout the city is a crucial factor to achieve this result. Unfortunately, we were not able to obtain reliable data to study the initial notification delay (i.e., the interval between the time that an accident happened and the time that the EMS center was notified). The delays are crucial to the eventual outcome of each dispatch. The mean of the scene-arrival-to-scene-departure interval was 12.85 minutes. It was not significantly different from the corresponding time interval of Seattle EMS system (i.e., 10 minutes) (19). It seems that, in our series, EMS personnel did not waste time to do unnecessary procedures at the scene of accident. Other valuable time interval, in our study, was the scene-departure-to-hospital-arrival interval. On average, this time was relatively high (i.e., 36.42 minutes). Road-related problems are significant factors affecting this time interval. The total mean dispatch-beginning-to-hospital-arrival interval was 55.42 minutes. Although there is no standard time for this time interval, it is significantly higher than the corresponding time for Seattle EMS system (i.e., 31 minutes, P = 0.015). (17)

### 5.4.Limitations

We should note that additional studies with larger sample size should be designed to analyze the associations more definitely. Our information about non-EMS transferred cases was limited. Moreover, we had no information about the time delay from calling an EMS until arrival at the scene.

### 5.5. Conclusions

Organized trauma systems have improved patient care in civilian gunshot victims. However, the majority of victims are transferred by non-ambulance vehicles. Also, the emergency care and hospitalization time of patients who were transferred by non-EMS was not different from others. The time intervals of Mashhad EMS dispatches, from beginning of a dispatch to arrival at the scene and transfer of the injured, were relatively acceptable. Nevertheless, increasing accessibility to organized trauma systems, efficient emergency road-traffic planning, equipping ambulances and training of personnel, particularly in private services, may improve prehospital management more efficiently.

## References

[1] Zafarghandi MR, Modaghegh MH, Roudsari BS (2003). Preventable trauma death in Tehran: an estimate of trauma care quality in teaching hospitals.. J Trauma..

[2] Zargar M, Modaghegh MH, Rezaishiraz H (2001). Urban injuries in Tehran: demography of trauma patients and evaluation of trauma care.. Injury..

[3] Consunji RJ, Serrato Marinas JP, Aspuria Maddumba JR, Dela Paz DA, Jr (2011). A profile of deaths among trauma patients in a university hospital: the Philippine experience.. J Inj Violence Res..

[4] Demetriades D (1997). Penetrating injuries to the thoracic great vessels.. J Card Surg..

[5] Harirchi I, Salehi M, Ghazisoltani M, Sanatkar-Far M, Satarzadeh R (2004). Gunshot wound of the heart with embolism to the right axillary artery.. Int Surg..

[6] Maiden N (2009). Historical overview of wound ballistics research.. Forensic Sci Med Pathol..

[7] Moon E, Kondrashov D, Hannibal M, Hsu K, Zucherman J (2008). Gunshot wounds to the spine: literature review and report on a migratory intrathecal bullet.. Am J Orthop (Belle Mead NJ)..

[8] (1995). Violence in America: a public health crisis--The role of firearms. The Violence Prevention Task Force of the Eastern Association for the Surgery of Trauma.. J Trauma..

[9] Volgas DA, Stannard JP, Alonso JE (2005). Current orthopaedic treatment of ballistic injuries.. Injury..

[10] Svenson JE (2000). Patterns of use of emergency medical transport: a population-based study.. Am J Emerg Med..

[11] Arreola-Risa C, Mock CN, Lojero-Wheatly L, de la Cruz O, Garcia C, Canavati-Ayub F (2000). Low-cost improvements in prehospital trauma care in a Latin American city.. J Trauma..

[12] Cales RH, Trunkey D. D. . (1985). Preventable trauma deaths: A review of trauma care systems development.. J Am Med Assoc..

[13] Shackford SR, Hollingworth-Fridlund P, Cooper GF, Eastman AB (1986). The effect of regionalization upon the quality of trauma care as assessed by concurrent audit before and after institution of a trauma system: a preliminary report.. J Trauma..

[14] Cornwell EE, 3rd, Belzberg H, Hennigan K, Maxson C, Montoya G, Rosenbluth A (2000). Emergency medical services (EMS) vs non-EMS transport of critically injured patients: a prospective evaluation.. Arch Surg..

[15] Demetriades D, Chan L, Cornwell E, Belzberg H, Berne TV, Asensio J (1996). Paramedic vs private transportation of trauma patients. Effect on outcome.. Arch Surg..

[16] Cornwell EE, 3rd (2003). Current concepts of gunshot wound treatment: a trauma surgeon's perspective.. Clin Orthop Relat Res..

[17] Arreola-Risa C, Mock CN, Padilla D, Cavazos L, Maier RV, Jurkovich GJ (1995). Trauma care systems in urban Latin America: the priorities should be prehospital and emergency room management.. J Trauma..

[18] Feliciano DV, Moore EE, Mattox KL (1996). Trauma..

[19] Bock FB, Berk WA, Banner SC, Wilson RF, Walt AS (1996). Prehospital medical care of the injured patient.. Management of Trauma..

